# Risk factors of postoperative pulmonary complications following elective craniotomy for patients with tumors of the brainstem or adjacent to the brainstem

**DOI:** 10.3892/ol.2014.2374

**Published:** 2014-07-23

**Authors:** HUI CHU, BIN-WEN DANG

**Affiliations:** Department of Respiratory Medicine, Beijing Tiantan Hospital, Capital Medical University, Beijing 100050, P.R. China

**Keywords:** brain tumor, craniotomy, postoperative pulmonary complications, risk factors

## Abstract

The aim of the present study was to analyze the risk factors of postoperative pulmonary complications (PPCs) of elective craniotomy for patients presenting with brainstem tumors or tumors adjacent to the brainstem. A total of 162 consecutive patients with a brainstem tumor or adjacent brainstem tumor undergoing elective craniotomy were included and monitored. Potential risk factors were identified by data collection and monitoring of the PPCs, as well as the performance of single factor analysis (using the χ^2^ test). In addition, the independent risk factors of PPCs were screened by logistic analysis. A total of 39 cases of PPC were included in the current study, with an incidence rate of 23.9%. The analysis indicated that smoking history, previous pulmonary diseases, an American Society of Anesthesiologists classification >II and partial tumor resection were risk factors of PPC following an elective craniotomy. Smoking history and partial tumor resection were identified to be independent risk factors of PPCs.

## Introduction

Postoperative pulmonary complications (PPCs) refers to the incidence of postoperative clinical manifestations of pulmonary abnormalities. These complications have a negative effect on disease progression, resulting in patients undergoing prolonged periods of hospitalization in addition to increasing the rate of postoperative mortality (1). PPCs predominantly include pneumonia, bronchitis, atelectasis, deterioration of lung disease and respiratory failure, which together account for 11.2–24.6% of all pulmonary-associated complications (2,3). Brainstem tumors refer to tumors that occur in the brainstem, including the midbrain, pons and medulla oblongata, and adjacent brainstem tumors refer to the tumors that occur in the area surrounding the brainstem, invading and oppressing the brainstem, and affecting brainstem function. A craniotomy for this type of tumor has different features when compared with other craniocerebral surgical procedures, which may be due to the specific location of the tumor, which results in a complex and risky procedure (4). Clinical findings of PPCs are well known, however, the incidence rate and associated risk factors are not often reported. Therefore, it is considered to be necessary to independently investigate this type of craniocerebral surgical procedure. The present prospective study was performed at the Beijing Tiantan Hospital (Beijing, China). This hospital includes a Neurosurgery Institute, in which the brainstem ward has 42 beds, predominantly for patients admitted for surgical intervention of brainstem or adjacent brainstem tumors, therefore, providing a large number of patients for inclusion in the present study. A prospective study was therefore conducted, which aimed to identify risk factors of PPCs that are commonly observed in patients following surgical intervention of brainstem tumors, and to facilitate with reducing the incidence of PPCs.

## Subjects and methods

### Subjects

A total of 162 consecutive patients with a brainstem tumor or adjacent brainstem tumor who underwent an elective craniotomy between August 2012 and February 2013, were included in the present study. This study was conducted in accordance with the Declaration of Helsinki and with approval from the Ethics Committee of the Beijing Tiantan Hospital, Capital Medical University (Beijing, China). Written informed consent was obtained from all participants.

### Methods

A senior physician of the Department of Respiratory Medicine performed all patient examinations and data collection. The director of the department was consulted to resolve any queries. Routine preoperative patient evaluations were conducted to obtain the following data: Patient age, gender, smoking history, body mass index, routine blood analysis, blood glucose and serum albumin levels, liver and kidney function, tumor location, imaging data and the American Society of Anesthesiologists (ASA) physical status classification grade. Postoperatively, the anesthetic methods and medicines, surgery duration, postoperative pathological diagnosis, tumor size and whether a full, near-total or partial tumor resection was conducted was recorded. Postoperative visits for seven consecutive days were performed to monitor the respiratory symptoms and body temperature; in addition, chest X-rays and blood analyses were performed when necessary. These data assisted the neurosurgeon with the diagnosis and treatment of the PPCs.

### Diagnostic criteria of the PPCs

PPCs were grouped into five categories as follows: (i) Pneumonia; classified as a recent pulmonary infiltration by chest radiography associated with at least two symptoms including, purulent tracheobronchial secretion, body temperature >38.3°C and >25% leukocytes in circulation above the base count (5,6). (ii) Tracheobronchitis; classified as an increase in volume, or a change in the color or purulent aspect of the tracheobronchial secretion with a normal chest radiograph (5,6). (iii) Atelectasis; classified by evidence from a chest radiography of a pulmonary atelectasis with associated acute respiratory symptoms (6,7). (iv) Acute exacerbation of chronic obstructive pulmonary disease (COPD); an event in the natural course of the disease characterized by a worsening of the patient’s baseline dyspnea, a cough and/or sputum, which is outside of the normal variability that is sufficient to warrant a change in treatment management. In cases where a chest radiograph indicated shadowing, consistent with infection, the patient was considered to have pneumonia (8). (v) Respiratory failure; characterized by a shortness of breath, dyspnea, respiratory frequency of >25 beats/min, with blood gas analysis (with no oxygen) revealing a partial pressure of O_2_ (PaO_2_) <60 mmHg and/or partial pressure of CO_2_ >50 mmHg, and an O_2_ inhalation oxygenation index (PaO_2_/fraction of inspired O_2_) ≤300 mmHg.

### Statistical analysis

The possible risk factors were statsitically analyzed using SPSS (SPSS, Inc., Chicago, IL, USA). The χ^2^ test was used for univariate analyses, and statistically significant factors underwent a logistic regression analysis to screen the independent risk factors of PPCs. P<0.05 was considered to indicate a statistically significant difference.

## Results

### Subject data

The present study included a total of 162 patients (males, n=70; females, n=92), aged 16–78 years, with a mean age of 44.33±1.02 years. Patients aged ≥60 and ≥65 years accounted for 9.26% and 1.85%, respectively of the total population. The patients exhibited normal preoperative hepatic function, routine blood test results, renal function, and serum albumin and blood sugar levels. No serious cardiopulmonary insufficiencies (ASA class >III patients, n=0; ASA class III patients, n=6; COPD patients, n=4; heart disease patients, n=2; senile valve disease patients, n=1; coronary heart disease patient, n=1) and no patients with a history of malignant tumors were identified. A total of 25 cases had a history of smoking and continued to smoke eight weeks prior to surgery.

### Distribution of tumor locations

There were 68 cases of brainstem tumors and 94 cases of adjacent brainstem tumors. There were 121 cases of benign tumors, 41 cases of malignant tumors, 59 cases of meningioma, 32 cases of neurilemmoma, 17 cases of glioma, 10 cases of chordoma, 10 cases of cavernous hemangioma, seven cases of hemangioblastoma, seven cases of ependymoma, six cases of astrocytoma, six cases of epidermoid cyst, four cases of glomus jugular tumor and four cases of other tumors.

### Anesthesia and surgical procedure

All patients underwent a craniotomy under intravenous anesthesia and tracheal intubation with mechanical ventilation, without the use of long-acting neuromuscular blocking agents, such as pancuronium bromide. All surgical procedures were performed by a team of three surgeons, following which the patients were admitted to the Department of Neurosurgery ward. The average surgery duration was 6.46±0.16 h (range, 3.00–12.25 h). A total resection was performed in 134 cases and a near-total or partial resection was performed in 28 cases.

### Pulmonary complications

There were 39 cases of PPCs, with an incidence of 23.9% (Table I).

### Single factor and logistic regression analysis

Single factor analysis demonstrated an association between PPCs and smoking history, underlying lung disease, ASA class >II and partial tumor resection. A logistic regression analysis on these risk factors indicated that smoking history and partial tumor resection were independent risk factors of PPCs (Tables II and III).

## Discussion

The present study has identified that, following elective craniotomy for patients with a brainstem or adjacent brainstem tumor, the incidence of PPCs was 23.9%. The occurrence of PPCs was significantly associated with smoking history, instances of previous pulmonary disease, ASA class >II and partial tumor resection. Smoking history and partial tumor resection were identified as independent risk factors of PPCs. To the best of our knowledge, this is the first report identifying partial resection as an independent risk factor.

The good, general condition of the patients that were enrolled in the present study indicates the uniformity of the population. There were 162 patients with normal preoperative hepatic and renal function, normal serum albumin and blood sugar levels, no serious cardiopulmonary insufficiencies (only six cases of patients with ASA class III, four cases of COPD and two cases of heart disease) and no histories of malignant tumors. All of the tumors were located in the narrow position of the brainstem or adjacent to the brainstem. All surgical procedures were performed under intravenous anesthesia, without the use of long-acting muscle relaxants and were performed at the same incision position by a team of three surgeons.

Due to the abovementioned reasons, the present study may avoid the confounding factors regarding PPCs and may, therefore, enable improved understanding of the associated risk factors of brain tumor patients with regard to PPCs. The present study has shown that partial tumor resection is an independent risk factor of PPCs, with subtotal and near subtotal resection surgery being associated with the highest incidence of PPCs. Thus, partial brainstem tumor resection has been identified as an independent risk factor of PPCs, which to the best of our knowledge, has not yet been reported in previous studies. This association may be due to residual tumor tissue, which continues to affect cerebral function. In addition, the present study has identified general risk factors of PPCs, including smoking history, which is a previously known independent risk factor of PPCs following lung, cardiac and abdominal surgery (9–15) and craniocerebral-associated surgical procedures (3). The present study demonstrates that smoking history is an independent risk factor of PPCs following brainstem and adjacent brainstem tumor surgery.

In contrast to previous studies (1,9,10,16–18), the present study has shown that PPCs are not associated with age or surgery duration, however, this may be due to differences in sample size or other factors. Clinical history of previous craniotomy procedures for brainstem and adjacent brainstem tumors is usually considered a contraindication for subsequent elective craniotomies. Although the procedure can be performed, the risk is high, therefore, strict screening of patients is required. Screening should include monitoring of the general characteristics of the patients; however, despite postoperative monitoring, the risk of developing PPCs remains high (24.1%). This is consistent with previous studies, which demonstrated that the likelihood of developing PPC following elective craniocerebral surgery is 11.2–24.6% (2,3).

Age is an independent risk factor of PPCs, which may be due to the age-associated decline of organ function. The data of the present study has shown that, despite variations in age, the function of each organ in the patients that were analyzed was normal, therefore, no significant correlation was identified between PPCs and age. Surgical procedures with a long duration have been considered to be an independent risk factor of PPCs. Qaseem *et al* (1) reported that the risk of pulmonary complications increases when surgery duration is >4 h. Sogame *et al* (3) reported that the probability of developing PPCs was significantly higher when surgery duration is ≥300 min when compared with procedures lasting <300 min. The present study showed that the surgery duration had no correlation with the development of PPCs following brainstem and adjacent brainstem tumor surgery. This may be due to the complexity of the surgery and a generally longer surgery duration. The mean surgery duration of patients in the present study was 6.06±0.16 h, whereas the previous studies have reported mean surgery times of 3, 4 or 5 h (6,17).

In conclusion, an ASA class >II and chronic lung disease have previously been identified as risk factors and independent risk factors of PPCs of chest, abdomen and other types of surgery, as well as risk factors and independent risk factors of PPCs of craniocerebral procedures (10,11,19–23). By contrast, in the present study, an ASA class >II and chronic lung disease were identified to be risk factors, although not independent risk factors, of developing PPCs following surgical excision of brainstem or adjacent brainstem tumors. The reason for this difference is unknown, however, may be associated with the small sample size, as an ASA grade >II was observed in only six cases (all were ASA grade III), and chronic pulmonary disease was observed in only seven patients. Thus, future studies are required to clarify these discrepancies by expanding the sample size.

## Figures and Tables

**Table I:** Type and incidence of PPCs in the cohort of 162 patients.

		Incidence, %	
		
PPC	Cases, n	Rate of total PPCs	Rate of overall number of patients
Pneumonia	20	51.3	12.3
Bronchitis	14	35.9	8.6
Acute respiratory failure	2	5.1	1.2
Primary pulmonary disease exacerbation	2	5.1	1.2
Atelectasis	1	2.6	0.6
Total	39	100	23.9

PPC, postoperative pulmonary complication.

**Table II:** Single factor analysis of risk factors of PPCs.

	PPC			
			
	Affected, n	Non-affected, n	χ^2^ value	P-value
Gender				
Male	21	49	2.368	0.124
Female	18	74		
Age, years				
>60	5	15	0.011	0.918
≤60	34	108		
ASA grade				
>2	5	1	11.971	0.001
≤2	34	122		
BMI				
>20	31	108	1.682	0.195
≤20	8	15		
BMI				
>25	14	36	0.610	0.435
≤25	25	87		
Tumor characteristic				
Malignant	13	28	1.750	0.186
Benign	26	95		
Location				
Brainstem	11	38	0.101	0.750
Adjacent to brainstem	28	85		
Tumor mass				
>100 g	7	19	0.138	0.711
≤100 g	32	104		
Partial resection				
Yes	12	16	6.534	0.011
No	27	107		
Surgical duration				
>5 h	31	82	2.307	0.129
≤5 h	8	41		
Smoking history				
Yes	12	13	9.258	0.002
No	27	110		
Basic lung disease				
Yes	4	3	4.377	0.036
No	35	120		

P<0.05 was considered to indicate a significant difference. BMI, body mass index; ASA, American Society of Anesthesiologists.

**Table III:** Logistic regression analysis on the independent risk factors influencing postoperative pulmonary complications.

Risk factor	B-value	Substandard error	OR value	95% CI	P-value
Partial resection	1.108	0.468	3.028	(1.210–7.574)	0.018
Smoking history	1.020	0.511	2.774	(1.019–7.555)	0.046

P<0.05 was considered to indicate a significant difference. B, binary logistic regression; OR, odds ratio; CI, confidence interval.
